# Christian Science

**Published:** 1910-05-07

**Authors:** Frederick Dixon

**Affiliations:** 23 & 24 Clun House, Surrey Street, Strand, W.C.


*To the Editor of* The Hospital.

### CHRISTIAN SCIENCE.

*To the Editor of* The Hospital.

Sir,—The notice of two pamphlets on Christian Science in your issue of the 16th inst. raises the controversy on that question once again. If denunciation would kill Christian Science, Christian Science would have died long ago. Denunciation has not killed Christian Science for two reasons: first, because it is invariably based on a complete misunderstanding of what Christian Science is contending for, and, secondly, because it is impossible to convince millions of people who have been healed themselves, or seen those dear or known to them healed, that the healing is nonsense. It seems to be forgotten that, incidentally, this very denial of healing is a serious aspersion on the capacity of the doctors who first treated and pronounced upon the cases. You cannot separate the two things in the minds of the audience to which the appeal is made. I know many doctors who manage to disagree with me, without regarding me as a knave or a fool. I, on the other hand, whilst differing root and branch from the regular practice of medicine, have persistently stated in public the recognition by the Christian Science Church of the devotion of the medical profession to the service of humanity as it judges right. Is there anything, therefore, to be gained by speaking of the "harpies who exploit it"—Christian Science—or "the monied nonentities who contribute to party funds"? If the writer knew what he was writing about he would know that the "harpies" have for the most part sacrificed their worldly prospects to endeavour to heal the sick according to their lights, whilst the "monied nonentities" simply do not exist. What he calls the party funds come from the subscriptions of the ordinary rank and file, including the "harpies," who feel this one of the ways in which they can show their gratitude for the health and happiness which they enjoy.

Yours truly,
Frederick Dixon.

23 & 24 Clun House,
Surrey Street, Strand, W.C.,
April 25, 1910.

